# Events as Power Source: Wireless Sustainable Corrosion Monitoring

**DOI:** 10.3390/s131217414

**Published:** 2013-12-17

**Authors:** Guodong Sun, Guofu Qiao, Lin Zhao, Zhibo Chen

**Affiliations:** 1 School of Information Science and Technology, Beijing Forestry University, 35 Tsinghua East Rd., Beijing 100083, China; E-Mails: sungd@bjfu.edu.cn (G.S.); zhibo@bjfu.edu.cn (Z.C.); 2 Key Lab of Structures Dynamic Behavior and Control (Harbin Institute of Technology), Ministry of Education, 25 Huaihe Rd., Harbin 150090, China; 3 School of Civil Engineering, Harbin Institute of Technology, 25 Huaihe Rd., Harbin 150090, China

**Keywords:** wireless sensor, corrosion monitoring, energy harvesting

## Abstract

This study presents and implements a corrosion-monitoring wireless sensor platform, EPS (Events as Power Source), which monitors the corrosion events in reinforced concrete (RC) structures, while being powered by the micro-energy released from the corrosion process. In EPS, the proposed corrosion-sensing device serves both as the signal source for identifying corrosion and as the power source for driving the sensor mote, because the corrosion process (event) releases electric energy; this is a novel idea proposed by this study. For accumulating the micro-corrosion energy, we integrate EPS with a COTS (Commercial Off-The-Shelf) energy-harvesting chip that recharges a supercapacitor. In particular, this study designs automatic energy management and adaptive transmitted power control polices to efficiently use the constrained accumulated energy. Finally, a set of preliminary experiments based on concrete pore solution are conducted to evaluate the feasibility and the efficacy of EPS.

## 1. Introduction

**Figure 1.**
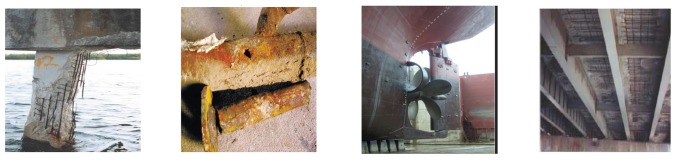
Some typical corrosion scenarios, respectively, in a steel-concrete structure, a pipeline, a cargo ship, and a marine platform (from left to right).

The corrosion, as shown in Figure 1, occurs in the structures involving steels or other kinds of metal. The corrosion of steel, referred to as the *Cancer of Steel*, has been a world-wide problem, which deteriorates the durability of structures and then degrades their serviceability [1], especially when the structures have been exposed to aggressive environments for a long time. Marine-based structures, such as ships, drilling platforms, bridge piers, *etc.*, are another major type of metallic structure subject to corrosion. Additionally, pipes that deliver water or oil are subject to corrosion, which attacks mostly from the inside. Corrosion-related failures and collapses may result in hydrocarbon releases and significant loss of production, as well as increased costs of maintenance, repair or replacement [1]. According to the statistics of World Corrosion Organization (WCO) [2], the annual direct cost of corrosion in the USA was a staggering $276 billion, or 3.1% of the gross domestic product (GDP) in 2001. Indirect costs to the user (such as society costs) are conservatively estimated to be equal to the direct costs. This means that the overall cost to society could be as much as 6% of the USA's GDP. Before appropriated protection and prediction strategies can be taken against corrosion, we have to first model and understand the corrosion status of structures. Therefore, the effective acquisition of corrosion data, as the first step of establishing accurate corrosion models, has attracted more and more attention in recent years.

So far, the main methods of capturing corrosion data have, however, been often followed by high costs in both deployments and human services. One common method uses the corrosion sensor powered by the grid or an external energy source (such as electric wires), which samples and returns data to end users via lines. Another method widely used is operated by people who use hand-held devices. Whenever the corrosion data is to be acquired, the hand-held device is firstly connected to the corrosion sensor that has been deployed in advance and then reads and stores the data for later analysis. Therefore, in corrosion monitoring applications, an effective and efficient way of sampling and acquiring data is highly needed and still a big challenge for long-term and human-free deployment.

With the development of embedded computing and wireless communicating techniques, wireless sensors have been utilized in a wide variety of applications [3], such as environmental monitoring, military surveillance and mobile targets tracking. In corrosion monitoring applications, for instance, the use of wireless sensors could lead to the easy deployment of sensing devices and real-time data acquisition, because the sensor is very small in size and no electric wires are needed to power the sensing devices and take back the data [4]. In particular, the sensor is often equipped with an MCU (Micro-Controller Unit), and consequently, local computation can be carried out, such that more efficient monitoring and controls can be achieved *in-situ*. Furthermore, the electrochemical essence of the corrosion process indicates that the techniques based on electrochemistry theory are the most direct and effective approaches to achieve the corrosion monitoring online. Electrochemistry-based corrosion monitoring means weak electric measurements. Therefore, the wireless sensors and networks are the appropriate and necessary platforms to carry on electrochemical methods in the field. Although the wireless sensor seems to be a good choice for low-cost, low-maintenance corrosion monitoring, one critical issue must be considered carefully: the sensor mote, often powered by battery, is extremely constrained in energy resource and then not able to provision for long-term operation (say five years, 10 years or longer) without battery replacements.

In this paper, we focus mainly on the wireless sensor-based corrosion-monitoring platform for RC (reinforced concrete) structures, which can achieve sustainable and autonomous operation, thereby satisfying the requirements of field experts for long-term and human-free monitoring. To this end, we design and implement a wireless sensor system, called EPS (Events as Power Source), which monitors the corrosion events in RC structures, while being driven only by the micro-energy released from the corrosion process; essentially, the corrosion energy not only is the event (field experts are interested in the dynamics of corrosion energy) but also serves as a power supply for EPS. In summary, the major contributions of this study are as follows:

- First, we build a sensing device to effectively detect corrosion events. It is small in size and able to output voltage signals, both of which make it easy to be physically connected with COTS (Commercial Off-The-Shelf) wireless sensor motes, such as the Telosb or Mica family motes.
- Second, we attempt to employ a low-cost COTS boost charger to harvest the micro-energy released in corrosive environments into a supercapacitor that can perpetually power the sensor mote as long as the corrosion continues, consequently removing the need for battery replacements.
- Third, to efficiently utilize the precious accumulated energy, we propose an adaptive scheme that runs on the MCU of EPS to schedule both the energy usage and the transmitted power of the sensor mote.
- Finally, we build a prototype of EPS and conduct a series of preliminary experiments, through which our designs are evaluated in terms of the feasibility and the efficiency of EPS.

The rest of this paper is organized as follows. We briefly introduce some significant related work in Section 2. Section 3 introduces the background of corrosion monitoring and some basics of the corrosion process that are important to our designs. Section 4 presents the detailed design of EPS in terms of the hardware platform and the communication schemes, and Section 5 conducts a preliminary test-bed experiment to evaluate EPS's feasibility. Section 6 concludes this study and introduces our future work.

## 2. The State of the Art

Energy-harvesting wireless sensor networks have attracted more attention recently. More effort is put into the design of an energy-harvesting circuit, which is often integrated to a COTS sensor mote, such as the Crossbow Mica-family mote and the Berkeley/Crossbow Telosb mote, and few effort into communication algorithms based on ideal energy-harvesting models, such as transmitted power control and routing.

The early work is attributed to Kansal *et al.* [5], who designed a solar-powered sensor node and an energy metric, EEHF (Environmental Energy Harvesting Framework), which assigns more loads at nodes with a higher energy-harvesting rate. Helimote [6] is an energy harvesting system with a single storage for buffering solar energy Built on a Mica2 mote, Helimote recharges two AA Ni-MH batteries, and it can learn its energy availability and usage via an energy-monitoring component. Jiang *et al.* [7] designed Prometheus, a hybrid energy storage system for solar energy. Based on Telos mote, Prometheus can be powered by the supercapacitors, called the primary energy buffer, or by the rechargeable Li-ion battery, called the secondary energy buffer. If the primary buffer energy is less than some threshold, the mote falls back to the secondary buffer until the primary one recharges fully again. Similar to Prometheus, AmbiMax [8] uses the hybrid energy storage. However, AmbiMax tracks the maximum power point automatically, without the control of MCU. Like our system, EverLast [9] is a supercapacitor-driven sensor mote and does not use any battery. EverLast uses a PFM (Pulse Frequency Modulation) controller and a PFM regulator to harvest the solar energy. To track the maximum power point, EverLast integrates a complex charging circuit. Besides outdoor solar energy, vibration, indoor light, thermal and wind energy sources have been studied to drive sensor motes [10,11]. The work in [12] gives a survey about harvesting vibration energy. Lee [13] designed an energy-harvesting chip for scavenging artificial light to power wireless mote. Wang *et al.* [14] presented some design considerations for an indoor light energy harvesting system. Yang *et al.* [15] studied the kinetic energy from a bike's counterbalancing movements and designed an electro-dynamic energy harvester. In [16], a novel energy source from tree movements is investigated, and a device using the force and displacement of a tree trunk is proposed to recharge a nickel hydride battery that powers a wireless sensor mote. There are a few works [4,17,18] that investigate only the generation principle of corrosion energy and suggest its feasibility, without system-level efforts. Until now, few attempts have been taken to accumulate the micro-electrochemical ambient energy to drive a wireless sensor mote for field application. Moreover, those above works mainly designed recharging circuitry for a given energy source. With the embedded techniques developing, however, richer recharging functions can be integrated into a low-cost COTS single chip, like BQ25504, the use of which is favorable to quickly build prototypes and then save the researcher's time to design more powerful, flexible software for the sensor mote.

Besides the platform designs, there are some studies that focus on the efficient communication strategies, aimed at improving the energy efficiency of energy-harvesting sensor systems. Based on an ideal energy-harvesting model, Lin *et al.* [19] developed a model to characterize the performance of multi-hop radio networks. A routing metric for energy-harvesting sensor networks is proposed in [20]; it uses hybrid energy storage and assigns different costs to the energy in the supercapacitor and the rechargeable battery; and it favors routes with more supercapacitor energy. Eu *et al.* [21] studied the optimal routing in an energy-harvesting sensor network with optimal relay node placement and investigated the impact of routing and node placement on the network performance. The authors in [22] investigated how to schedule the links and flows in energy-harvesting wireless networks to avoid the co-channel conflict, and [23] described a routing framework based on duty cycling for energy-harvesting sensor networks; the works in [21–23] just proposed algorithms for a general energy-harvesting sensor network with ideal models and do not focus on the sensor mote platform driven by the extremely-low output power (such as the corrosion energy).

The energy released from corrosion in RC structures are investigated in [4,18,24,25], where the energy, in the form of either voltage or current, is only analyzed as a kind of signal from which the corrosion status can be identified and estimated. The energy results presented in those works suggest the feasibility of using the corrosion energy to charge low-power sensor motes.

## 3. Background and Motivation

### 3.1. Electrochemical Energy Source

**Figure 2.**
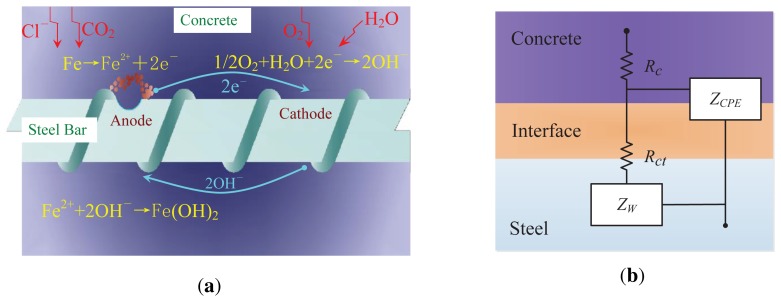
The basic principle of the steel's corrosion process [4] with the deficit representing the corrosion (**a**) and the equivalent circuit (**b**).

The fundamental factors that lead to the corrosion process in environments with steel are shown in Figure 2a, where the rust formation and the loss in cross-section both occur [24,26]. Generally, we consider steel's surrounding as a solution with some ideal ions. In reality, H_2_O, CO_2_ and CI^−^ are the fundamental elements contributing to corrosion. In Figure 2, CO_2_ and CI^−^ result in the general corrosion and the pitting corrosion, respectively. During the general corrosion process, the Fe atom is oxidized to Fe(OH)_2_, Fe_3_O_4_, Fe(OH)_3_ or Fe_2_O_3_ · *n*H_2_O at the anode region, while the O atom is reduced to O^−^_2_ at the cathode region [24]. In conclusion, the corrosion process of steel is essentially an electrochemical reaction process, which releases micro-energy; and the electrochemical potential (in millivolts), as well as the current (in milliamps) are often treated as the event signals from which the corrosion degree can be recognized accurately.

Since the electrochemical reaction of corrosion releases current, we can use an equivalent circuit shown in Figure 2b to describe the energy delivery in corrosion. The equivalent circuit is a universal transfer function to simulate and characterize the electrochemical features of the steel-solution system. In Figure 2b, *R_c_* and *R_ct_* measure the resistance of the solution and the reactive resistance of corrosion, respectively. *Z_W_* represents the diffusion impedance of O_2_ and *Z_CPE_* the dispersion impedance of the solution-steel interface. The corrosion characteristics can be identified and modeled once the elements in the equivalent circuit are confirmed. The parameters, *R_c_*, *R_ct_*, *Z_w_* and *Z_CPE_*, can be extracted accurately by fitting the experimental results at the time region. The authors of [18,24,25] presented the calculation of these parameters, which are beyond the scope of this study. The corrosion principle in Figure 2 is deduced from the corrosion in RC structures, but the corrosion in the marine or pipeline environment involving steels agrees with the identical principle.

### 3.2. Motivation

Corrosion is not only associated with societal issues (including human life and safety, the cost of corrosion and conservation of materials [27]), but is also a difficult phenomenon to understand, because of the lack of real-time corrosion data. So far, it is still a key challenge to efficiently acquire corrosion data without much human intervention and extra servicing costs.

A significant observation is that the electron is presented and transferred inside the barrier layer or on the surface of the reinforcing steel. The energy from the steel-smelting process is delivered by the electrochemical reactions and the physicochemical processes. Therefore, besides serving as the event signals, the corrosion energy brings an appealing opportunity to be employed as a kind of sustainable energy source to power the sensing device. Although the corrosion energy is able to supply a wireless sensor mote, two critical issues have to be addressed for practicality: (1) what device to use to sense the corrosion signal, as well as to output the corrosion energy; and (2) how to design an effective energy management policy, such that the wireless corrosion-monitoring sensor system can operate in a human-free fashion for a long-term period, like, 20 years, 30 years, or longer.

## 4. Designs

The main goal of EPS is to provide a sustainable solution to the corrosion monitoring application that is based on wireless sensors. The major challenges are designing a device for detecting corrosion events, accurately monitoring the corrosion status and using the harvested micro-corrosion energy. In the following sections, we first introduce the requirements of a real scenario and the architecture of EPS and then present our design details, component choices and implementation considerations.

### 4.1. Overview of Application and EPS Design

**Figure 3.**
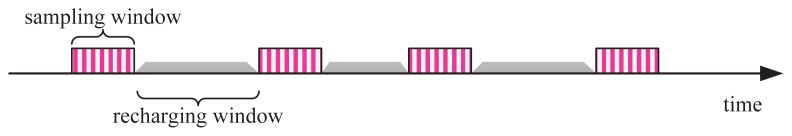
The operation series of EPS (Events as Power Source) with time increasing.

In corrosion monitoring applications, events *(i.e.*, the corrosion processes) are continual, slow and inconspicuous. Different from a mobile target tracking sensor system, which is a typical real-time scenario with almost a full duty cycle, it is reasonable and sufficient for corrosion monitoring applications to acquire short-term data, with a large time interval, such as one day or several days. Therefore, we can configure corrosion monitoring with a very small duty cycle and a short duty duration. In practice, for instance, it is sufficient that the sensor mote samples the corrosion environment once per day and 100 s every time; in other words, if EPS runs with a duty cycle of 0.12%, the application fidelity will not be deteriorated. Besides the duty characteristics, however, we have to consider the problem of matching the differences among the corrosion-sensing device, the charging unit, and the sensor mote, because they have different operational behaviors in voltage and output power. Figure 3 illustrates the operation series of EPS, where its operating times are divided into two types: the *sampling window* and the *recharging window*. In a sampling window, EPS samples sensors at a given frequency and transmits the reading once a sample is completed, while in a recharging window, EPS disables all application-specific tasks and recharges its energy buffer (a supercapacitor) until a specified supply voltage is achieved. As shown in Figure 3, EPS works with best efforts (sampling alternates, recharging continuously, as long as the corrosion is in progress). In addition, the time periods of different sampling windows can be kept similar by well-controlled energy management (to be discussed in a later section), while the recharging windows are time-varying, because the energy released from corrosion processes is rather dynamic in the time domain.

**Figure 4.**
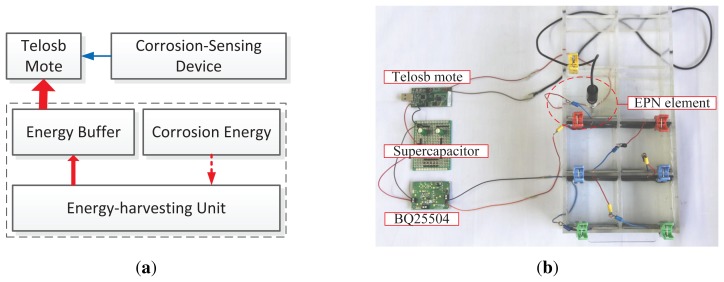
The architecture of the EPS sensor mote shown in (**a**) and the testbed with the concrete pore solution in the box shown in (**b**). EPN, electrochemical potential noise.

In EPS design, we build a three electrode-based corrosion-sensing device and an energy-harvesting unit, both of which connect to a Telosb mote (the architecture of EPS is shown in Figure 4a). We select Telosb mote, because it is: (1) equipped with a low-power MCU and a radio chip; (2) low-cost and size-small; and (3) easy to extend to support more external sensors via its multiple ADC (Analog-to-Digit Conversion) ports. Since the duty cycle of corrosion monitoring applications is extremely low and the corrosion energy always exists, we do not need a large energy buffer, and then, we use only a small-capacitance supercapacitor to store the corrosion energy, rather than using the hybrid energy storage system [7,10] or the rechargeable Li-battery [5,28], thereby reducing the deployment cost, the package size and the complexity of management.

### 4.2. Corrosion-Sensing Device

The electrochemical corrosion status of the steel-solution system can be recognized by an active monitoring technique (AMT) and a passive monitoring technique (PMT). According to the universal transfer function for the general corrosion in Figure 2, abundantly actuating signals can be used to excite the steel-solution system in the linear region and the nonlinear region. Therefore, the electrochemical elements, including *R_c_*, *R_ct_*, *Z_W_* and *Z_CPE_*, can be extracted in the time domain or in the frequency domain via nalyzing the responses of the steel-solution system to these actuating signals. For the pitting corrosion caused by CI^−^, the electrochemical emission spectrum (EES) in PMTs is the most effective technique to recognize the corrosion status.

**Figure 5.**
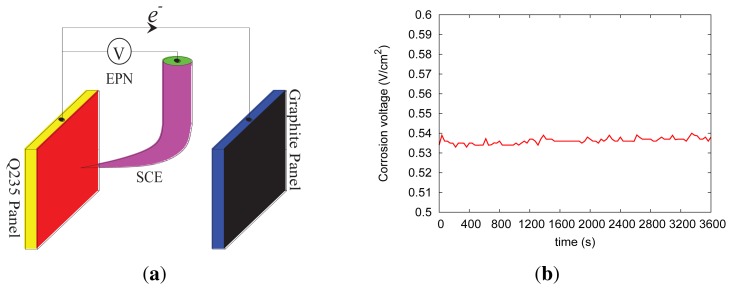
The three-electrode corrosion sensing device, where (**a**) and (**b**) show the schema and the open-circuit output voltage of the device, respectively. SCE, saturated calomel electrode.

The classical three-electrode system illustrated in Figure 5a is established to obtain the electrochemical potential noise (EPN) information of EES. The Q235 carbon steel, the graphite panel and the saturated calomel electrode (SCE) are used as the working electrode, the counter electrode and the reference electrode, respectively; such a three-electrode system is embedded in RC structures in practice or immersed in the simulated corrosive pore solution in lab experiments. It can be seen in Figure 5b that the open-circuit voltage output by the proposed sensing device ranges from 0.5 to 0.6 V, which demonstrates the feasibility of recharging the sensor mote. There are a few corrosion-detecting sensors that consider the trade-off between the package size and the energy-releasing capacity [4]. To clearly illustrate the mechanism of the corrosion-sensing mechanism, we adopt the simple three-electrode system here. EES, also named the fingerprint of pitting corrosion, exposes the trace of the pitting corrosion. Actually, EES is the only significant information directly related to the pitting corrosion, which can be monitored during the corrosion process in the field. EES essentially reflects the intrinsic information of the initial, metastable, repassivation and stable stages during the pitting corrosion process. With passive listening in to the reinforcing steel, confiding how the corrosive mediums intrude into the RC structures, the pitting corrosion status can be identified qualitatively via analyzing the EPN data in the time domain, the frequency domain, the wavelet domain or the chaos domain [25]. Considering the scope of this paper, here we do not discuss the approaches of analyzing corrosion data in detail.

### 4.3. Energy-Harvesting Unit

One goal of EPS is to drive the wireless sensor mote by harvesting micro-corrosion energy, without extra maintenance cost. In this section, like EverLast [9], we use only the supercapacitor to store the corrosion energy and power the Telosb mote. A supercapacitor is an electrochemical, small-package capacitor that offers very high capacitance and quick recharging. With superior cycle lifetimes, supercapacitors can allow greater than 500 thousand recharge cycles, approximately 1,000 times that of rechargeable batteries. A method of determining an optimal supercapacitor is introduced in [7], where the supercapacitor that maximizes the discharging period while keeping the output voltage above the minimum operating voltage is treated as the best. Note, additionally, that the higher the capacitance of a supercapacitor, the longer the time that is needed to fully recharge it. In our energy harvester, we choose a 2.7 V/10 F supercapacitor to store the corrosion energy, which is sufficient to drive the Telosb mote, whose working voltage ranges from 1.8 to 3 V. If the application-specific task requires dense computation and communication, a larger energy buffer could be chosen. In fact, we can connect multiple supercapacitors in series to obtain a higher voltage output or connect them in parallel to obtain a higher capacitance; of course, that leads to extra circuits and costs, as well as a bigger package. For the corrosion monitoring application, a single 2.7 V/10 F supercapacitor is a desirable choice.

The current draw of the Telosb mote in active state is 23 mA with the radio chip on, and that in sleep state is just 5 *μ*A [29]. For an ideal capacitor, Equation (1) gives its discharging duration with the voltage drop from *V*_1_ to *V*_2_, where *C* is its capacitance and *I* is the working current. Suppose here that the initial voltage of the 10 F supercapacitor is 2.5 V and the Telosb mote works in active state until the supercapacitor's voltage drops to 2.0 V. Then, we know that the Telosb mote's operation will last three minutes. If the task is duty-cycled, say 10%, then the mote will work for 30 min before the supercapacitor's voltage reaches 2.0 V, if the energy consumption of the sleep state is not taken into account.

(1) $$\Delta T=\frac{C\left(V_{1}-V_{2}\right)}{I}$$

The corrosion energy released from the corrosion process in RC structures is very low in power: the output voltage and current are often orders of hundreds of millivolts and hundreds of microamps (or even tens of microamps), respectively. Thus, such a power level is not sufficient by itself to recharge the supercapacitor and then cannot feed the Telosb mote. To address this practical issue, we use TI's BQ25504 [30], an efficient COTS boost charger, to connect the micro-energy source and the supercapacitor. The BQ25504 unit, which costs a few USA dollars, is able to harvest the nano-level of power and can efficiently boost the input to 3.2 V by default. It needs at least 330 mV of input in the cool start stage, and after cool-starting, the least input voltage needed is just 110 mV. For avoiding overcharge to the 2.7 V supercapacitor, a diode of 0.6 V can be used to cap the BQ25504's output voltage into 2.6 V, which can still drive the Telosb mote successfully.

### 4.4. Scheduling Schemes

To efficiently utilize the micro-energy accumulated from the corrosion process, it is necessary and important to determine a set of control logics running on Telosb's MCU; in fact, the wireless sensor mote that EPS depends on overwhelms the traditional sensors with its computation ability, which provides an opportunity to implement the automatic *in-situ* management of EPS in terms of energy usage and communication. In this section, we first introduce a method of estimating the application-specific energy budget and then propose a simple-but-effective, scheme to manage the recharging and the discharging of the supercapacitor, such that the application fidelity can be met. Finally, we use a link-quality-adaptive transmission power control scheme to further reduce the energy consumption.

#### 4.4.1. Modeling the Discharging Ability

Before determining a schedule of the harvested energy, the first step is to understand the discharging profile of the supercapacitor in use well. Equation (2) is the commonly-used expression calculating the discharging energy (in joules) over a voltage drop from *V_S_*(0) to *V_S_*(*T*), where *V_s_*(*t*) represents the voltage in volts at time *t*. In practice, however, *Vs*(*T*), the ending voltage of the supercapacitor, cannot be determined accurately *a priori*, especially when *T* is relatively large, because it depends not only on the the actual load (the current need), but also on the supercapacitor itself. To this end, Equation (2) can be rewritten as Equation (3), where only the current load (*I_active_*) in Telosb's active modeis considered.

(2) $$E(T)=\frac{1}{2}C(V_{S}{\left(0\right)}^{2}-V_{S}{\left(T\right)}^{2})$$

(3) $$\int_{0}^{T}I_{\text{active}}\times V_{S}(t)dt$$

(4) $$\begin{array}{cc} \approx I_{\text{active}}\overset{k}{\underset{i=1}{\text{∑}}}V_{S}(i\times \Delta t) & \;\left(k=\frac{T}{\Delta t}\text{and}\;0<\Delta t\leq T\right) \end{array}$$

**Figure 6.**
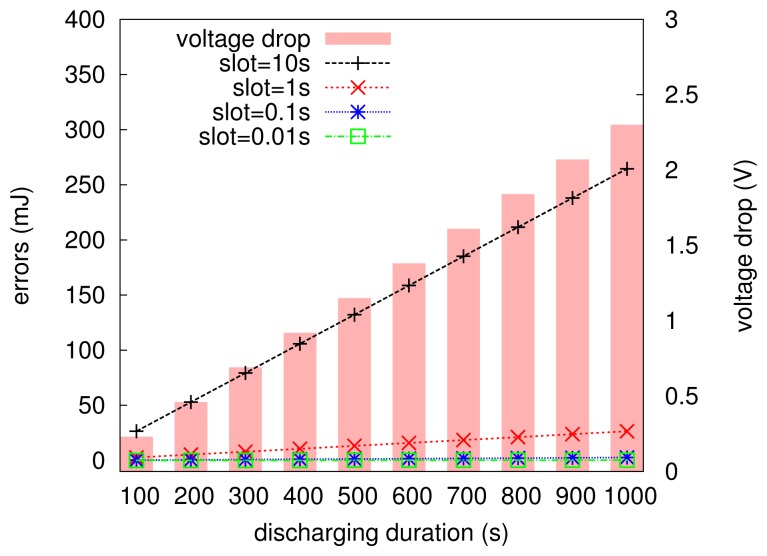
The voltage drops and the distributions of errors over different discharging times. The initial voltage is 2.6 V and the current is 23 mA in this numerical analysis. In this figure, Δ*t* is renamed by slot.

The MCU in the embedded computing scenario is very constrained in computation ability; for example, the MSP430, used in the Telosb mote, is a 8 MHz/16 bit processor, and Atmega, another widely-used MCU family, is a 8 MHz/8 bit processor. In general, low-cost MCUs cannot support such complicated integration calculation shown in Equation (3). To this end, we approximate the calculation of Equation (3) with a summarizing expression given in Equation (4). It is clear that Equation (4) is equivalent to Equation (3), only when Δ*t* tends to be zero. However, the MCU is hard to or even cannot deal with too precise numbers, say, 10^−8^, which is often used to approximate zero in a computer system. Thus, a relatively larger Δ*t* should be chosen in practical programs for the ease of computation. Fortunately, the approximation does not experience significant errors for low-power embedded systems. Figure 6 numerically plots the errors resulting from Equation (4), where the current value is 23 mA, the initial voltage of the capacitor is 2.6 V and the voltage drop after each Δ*t* is calculated according to Equation (1) (here, the supercapacitor is assumed to be ideal in a short period of time, say one second). It can be seen that the increment of discharging time leads to the larger voltage drop of the capacitor. We found that when Δ*t* (slot) is set to be 10 s, the error (in units of millijoules) is very significant; whereas when it is set to be 1 s or less, the errors increase very slowly with the charging duration increasing, and they are only the order of tens of millijoules. In particular, the setup of Δ*t* with 1 s, 100 ms or 10 ms will not lead to distinct errors; however, it is clear that Equation (4) needs less computation with Δ*t* set to be 1 s than that with Δ*t* set to be 0.01 s. Therefore, Δ*t* is set to be one second in EPS to estimate the energy budget over the time period of *T*.

#### 4.4.2. Control of Discharging and Recharging

What the field experts are interested in is the corrosion events (physical or chemical signals); they determine the corrosion degree by using the information extracted from those event data. Therefore, they have a specific demand on the data profile, including the rate of data (sampling frequency), the quantity of data and the accuracy of data. For corrosion monitoring, experts, the observers and users of data usually need a set of event data that is acquired over some period of time with a specified frequency. For instance, with a 2 Hz sampling rate, the corrosion data within a continual five minutes will achieve the accurate identification of the local corrosion degree. Next, we will introduce the design of energy scheduling by a walk-through example with the above parameters.

For the 2 Hz sampling task that lasts five minutes, a total of 600 samples will be carried out on the ADC interface by the MCU. In detail, a sample will experience three stages before it can be received by the base station. First, it is read from the ADC interface and converted to a reading in engineering units; second, the reading is processed by the MCU if necessary; third, a packet is generated involving the reading and then delivered to the radio chip for transmission. For the Telosb mote, those three stages can be completed within 40 ∼ 60 ms. For the simplicity of analysis, now, suppose an entire event sample needs 100 ms; then, completing 600 samples needs 60 s, within which the sensor mote has to work with the current of *I_active_*. Moreover, if the starting voltage is set to be 2.6 V and *I_active_* (the current of the active mode) 23 mA, we can then know that the total energy demand is 3.49 J, and the ending voltage is 2.46 V, according to Equation (4). Note here that we neglect the energy consumption of EPS in sleep mode.

The above analysis indicates a method that establishes a map from the specification of event data to the energy demand and then to the ending voltage, by which we can schedule the discharging and recharging process of EPS. We set two voltage bounds, *V*_1_ and *V*_2_, both of which are within the lowest and the highest supply voltages of the supercapacitor. Once the supercapacitor's voltage is beyond *V*_1_, EPS can start the sampling task; and it will have to stop the task when *V*_2_ is reached. For the example discussed previously, *V*_1_ and *V*_2_ are 2.6 V and 2.46 V, respectively. If some new task should be updated according to the user's requirement, only the adjustment of those tow voltage bounds will obtain a proper energy budget, without any complex computation.

**Figure 7.**
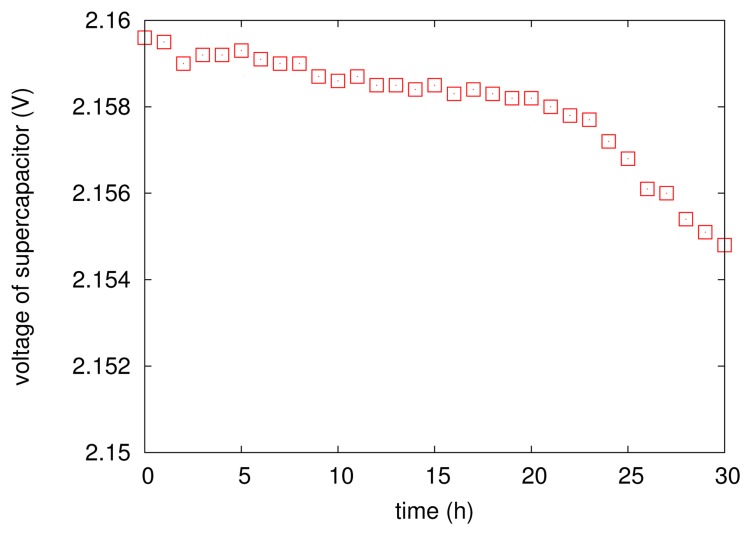
The voltage variation after disconnecting the energy source from the supercapacitor.

It is worth noticing that the above energy scheduling policy does not consider the leakage of the supercapacitor. Actually, the leakage of the capacitor has been mentioned in literally a lot of previous work. However, there was no evidence that shows that the leakage in EPS has impacted the operation of the proposed scheduling. We conduct a controlled experiment: during the EPS's running, we disconnect the corrosion energy source from the supercapacitor for 20 h. Figure 7 illustrates the variations of supercapacitor's voltage during the period of disconnection. It is fairly clear that the supply voltage of the supercapacitor seems more or less unaffected: the voltage drop (leakage) is just 2 mV within 20 h. The insignificant leakage of the supercapacitor in EPS simplifies the calculation of Equation (4) and then guarantees the accuracy of energy management.

#### 4.4.3. Link-Quality-Adaptive Transmitted Power Control

In EPS, the sensor mote transmits its data to a base station, another Telosb mote connected to a PC, where all data is stored for further analysis. As we know, the energy is the overriding constraint for a wireless sensor system; and in reality, the most energy-hungry component is the radio chip, which draws much greater power to perform communications, in comparison with other components. One major method of saving energy in communications is to reduce the transmitted power. For the Telosb mote, there are eight discrete choices for configuring the transmitted power, from 0 dBm down to –31 dBm. However, the lower the transmitted power, the lower the link quality, which is a metric for the delivery reliability of a link. Additionally, the distances from sensor motes to the base station may be different. It is not necessary for the motes that are not far away from the base station to use a great deal of transmitted power; it is another space for saving energy. In summary, the tradeoff between the energy savings and the guarantee of reliability should be considered carefully in the corrosion monitoring environment. In this section, we design a light-weight link-quality-adaptive scheme to adjust the transmitted power, such that the energy consumption is the least possible, while maintaining a high-quality (reliable) wireless link between the sensor mote and the base station.

The proposed transmitted power control involves two stages: find the minimum feasible transmitted power and adjust the transmitted power for good link quality with time increasing. Here, let *P_tx_* = {*p*_1_, *p*_2_, …, *p_m_*} be the set of all possible transmitted powers the sensor mote can support, where *P_i_* < *p_i_*_+1_ (1 ≤ *i* ≤ *m* − 1). For stage one, the mote continues to send a set of probe messages to the base station, with an incremental transmitted power, until it can receive almost all acknowledgments (ACKs). For example, if the mote with a transmitted power of *p_i_* cannot receive any ACKs, it then continuously increases its transmitted power to *p*_*i*__+1_, *p*_*i*+2_, up to *p*_*i*+*k*_ (1 ≤ *k* ≤ *m* − *i*), until all ACKs can be received correctly After this stage, the sensor mote establishes a reliable link to the base station at that time. For stage two, the main task is to maintain the link quality by adjusting the transmitted power when some undetermined factors affect the link.

Before maintaining the link quality, how to measure the link quality should be considered firstly In general, the PRR (Packet Reception Rate) is the best desirable metric for link quality. For instance, if eight of ten packets over a link are responded to with ACKs, the link PRR is 80%. The PRR metric is so much more accurate than the RSSI (Received Signal Strength Indicator) or the LQI (Link Quality Indicator), both of which are calculated by the radio chip itself at the packet level to estimate the link quality. In comparison with RSSI and LQI, the high accuracy of PRR is achieved at the expense of sending a set of consecutive probe messages, which needs the extra bandwidth and energy of the wireless mote. Fortunately, the wireless channel in sensor networks will experience no significant dynamics during a short period of time, say, tens of minutes [31], especially since each time window of sampling in the corrosion monitoring application is often less than half an hour (the time window of five minutes is sufficient, in practice). Therefore, EPS can reasonably use PRR to measure the link quality. Moreover, EPS employs the piggy-backing of packets to obtain the PRR without using any probes, thereby almost needing no extra communication cost.

(5) $$\begin{array}{cc} p_{i+1}=p_{i}⊕1 & \;1\leq i\leq m-1 \end{array}$$

(6) $$\begin{array}{cc} p_{i-1}=p_{i}⊖1 & \;2\leq i\leq m \end{array}$$

Algorithm 1 presents the details of controlling the transmitted power. For many sensor applications, like our corrosion monitoring, the loss of a small fraction of packets may be tolerated; thus, we set a threshold of link quality, denoted by *Thr_prr_*, beyond which a link is treated as reliable. For EPS, *Thr_prr_* is set to be 90%. A single sampling time window is five minutes with a 2 Hz sampling rate. For the series of packets in a sampling time window, we use the first *m* successive packets as probes, and it is easy to know the PRR according to the number of received ACKs. If the PRR < *Thr_prr_*, we then increase the transmitted power with one level, that is, from *p_i_* to *p*_*i*+1_. This process is repeated until the PRR satisfies the requirement; thereafter, the mote will use the updated transmitted power to send out packets until the current sampling time window has reached timeout. In Algorithm 1, for simplicity, we use two expressions in Equations (5) and (6) to represent the increasing and decreasing of the transmitted power with one level, respectively.

(7) $${\text{Thr}}_{\text{prr}}\leq \frac{N-l\times m}{N}$$

Lines from 4 to 8 of Algorithm 1 describe how to increase the transmitted power if the firstly measured PRR is not beyond threshold *Thr_prr_*, while lines from 10 to 15 deal with the situation in which too high of transmitted power is used in previous sampling windows. *K* at Line 10 is empirically set to reflect the fact that the link dynamics itself involves the process of quality improvement. In our test-bed, *K* is set to be one. Another factor that should be considered is *m*, the number of probe messages. For a single sampling window within which a mote will generate *N* packets, the number of packets received by the base station is not allowed to be less than *Thr_prr_*; otherwise, the transmitted power control will fail; Equation (7) gives a way of estimating the *m* of Algorithm 1, where *l* is the number of attempts of increasing the transmitted power. Obviously, a larger *m* will waste the bandwidth and the energy of the sensor mote, but it may lead to a smaller *l*, while a small *m* cannot estimate the link quality accurately and, then, may need the transmitted power increased more times. We empirically set *m* to be 10 for our prototype according to Equation (7). Notice that we use an exponential average weighted method to update the PRR in Line 7, in order to achieve a more stable PRR and then the more reliable delivery.



**Algorithm 1** Link quality adaptive transmitted power control.
**Require:** the initial feasible transmitted power, *p_init_*, the currently maintained transmitted power, *p_curr_*, the link quality threshold, *Thr_prr_*, and the incremental step, *K*.**Ensure:** the last updated transmitted power, *p_curr_* 1:Send out *m* successive packets and receive *n*_1_ ACKs 2:
$\text{PRR}\leftarrow \frac{n1}{m}$ 3:**if** PRR ≤ *Thr_prr_*
**then** 4: **while** PRR ≤ *Thr_prr_*
**do** 5:  *p_curr_* ← *p_curr_* ⨁1 6:  Send out *m* successive packets and receive *n*_2_ ACKs 7:  
$\text{PRR}\leftarrow \alpha \times \text{PRR}+(1-\alpha )\times \frac{n_{2}}{m}$ 8: **end while** 9:**else** 10:  **if** (*curr* — *init*) ≥ *K*
**then** 11.  *p_curr_* ← *p_curr_* ⊖ 1 12:  Send out *m* successive packets and receive *n*_3_ ACKs 13:  
$\text{PRR}\leftarrow \frac{n_{3}}{m}$ 14:  Go to line 4 15: **end if** 16:**end if** 17:Send out the remaining packets with transmitted power of *p_curr_* 18:**return**


## 5. Experimental Results

We build a small-scale prototype of EPS based on the Telosb mote and the TinyOS programming environment, which is shown in Figure 4b, and then, we evaluate the feasibility and efficiency of EPS by preliminary experiments. We use 3% NaCl concrete pore solution to simulate the corrosive environments of RC structures in which the steel will easily experience corrosion. This simulation of the corrosive environment is reasonable, because the densities of chloride in the sea and in concrete structures are both around 3%. Many corrosion recognition studies are also evaluated using 3% or 3.5% NaCl solution [24,26]. For the energy-harvesting unit, we use a BQ25504 evaluation module to connect a 2.7 V/10 F supercapacitor and the corrosion-detecting device, which releases electric energy the moment corrosion occurs. A Telosb mote equipped with built-in temperature, humidity and light sensors is powered by the supercapacitor; besides the built-in sensors, the Telosb mote monitors the supercapacitor's voltage and the reference electrode that is embedded in the solution and outputs voltage signals. The voltage bounds, *V*_1_ and *V*_2_, are set to be 2.4 V and 2.2 V, respectively, that is, the mote can start the tasks of sensing and sending only when its supply voltage, *V_S_*, is beyond *V*_1_, and it has to stop all tasks, except maintaining a lightweight periodic *V_S_* checker, once the *V_S_* decreases to or below *V*_2_.

In our experiments, we first profile the supercapacitor in terms of the time of recharging period, which demonstrates the feasibility of the proposed energy-harvesting system for corrosion monitoring, and then, we evaluate the transmitted power scheduling scheme, which will measure the energy efficiency of our prototype.

**Figure 8.**
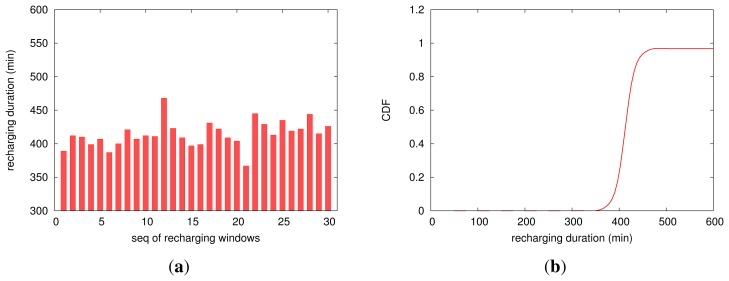
The distribution (**a**) and the CDF (**b**) of the time for the recharging periods.

Controlled by the two bounds for the supply voltage, *V_S_*, the mote will enter the recharging process once *V_S_* reaches *V*_2_; consequently, it needs a period of time to recharge the supercapacitor with the weak corrosion energy until *V_S_* increases up to *V*_1_. Therefore, the mote works intermittently, and we call each process of recharging a *recharging window* and the time needed by a recharging window the *recharging duration*. Figure 8 profiles EPS's recharging process from a mote (two motes are used in our experiments, but we report only one of them, because their results are very similar). Figure 8a plots the distribution of a successive 30 recharging windows, which shows that for running every task of sampling and transmitting, it will take several hours to fully recharge the supercapacitor; the recharging duration ranges from 367 to 468 min, with the average of 414.4 min and with the standard deviation of 19.4 min. According to Figure 8b, it can also be found that most of the recharging windows need a period of time between 400 and 430 min. We analyze the variation of the recharging duration from the point of view of electrochemical reactions resulting from the corrosion events. An aggressive anion, such as C1^−^, is able to enhance the flux of cation vacancies through the barrier layer. Under favorable conditions (voltage, pH, [C1^−^]), the vacancy condensation will occur at the metal-barrier layer interface, and hence, passivity breakdown will ensue. Therefore, the fresh surface of the metal will generate EPN transients. Furthermore, the shielding effect of the corrosion products can lead to the slight fluctuation of the corrosion currents. Therefore, the recharging duration of the supercapacitor presents a little bit of discreteness. It is worth noticing here that for different settings of supply voltage bounds, a different recharging duration will be needed. Therefore, in practice, we can adjust these two bounds according to the trade off between the recharging time and the application-specific loads.

**Figure 9.**
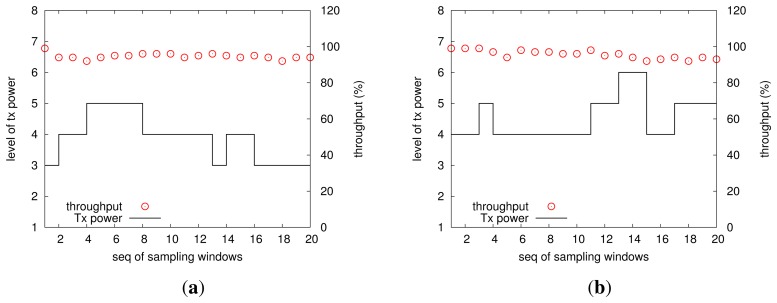
The transmitted power levels used by two motes, where (**a**) is for mote I; and (**b**) for mote II.

To further save the precious energy accumulated from the corrosion process, this study proposes a link-quality-adaptive control scheme for wireless transmissions, which is very energy-hungry for a wireless sensor mote. We examine the strength of our transmitted power control by controlled experiments in office environments, in which two motes send packets to the base station: one (mote I) is 1.5 m away from the base station and the other (mote II) 4 m away from the base station, and the link quality threshold is set to be 90%. The results of mote I and mote II are shown in the left and the right parts of Figure 9, respectively. Upon initialization, both motes will increase their transmitted powers from the lowest level until they can obtain a link quality beyond 90%, and we call the obtained transmitted power the *initial feasible transmitted power*. It can be seen that the initial feasible transmitted power levels for mote I and mote II are three and four, respectively (recall that the Telosb mote has eight discrete transmitted power levels). We know indoor environments (especially the office) bring more complex and random impacts to wireless communications [32,33]; moreover, the motes in use communicate with an IEEE802.15.4 2.4 GHz radio chip, which is low-power and of a low rate in the ISMband, thereby being more error-prone. During the operation of our experiments, a few students worked in the office of a size of 2.5 x 5 x 2.7 *m*^3^, and they often moved intentionally. The distribution of circles in the two sub-figures of Figure 9 illustrates that for all sampling windows, the link quality is not less than 90% configured *a priori*, thereby testifying to the feasibility of our transmitted power control. Additionally, it is clear that both motes adjust their transmitted powers, by either increasing or decreasing operation, in order to achieve the link quality threshold, while saving energy. It can be found that most of time, two motes use the initial feasible transmitted power to communicate, unless the current link encounters deterioration. The transmitted power adjustments, on the other hand, show the dynamics of wireless links and suggest the necessity of considering how to reduce the energy consumption in wireless communications.

**Figure 10.**
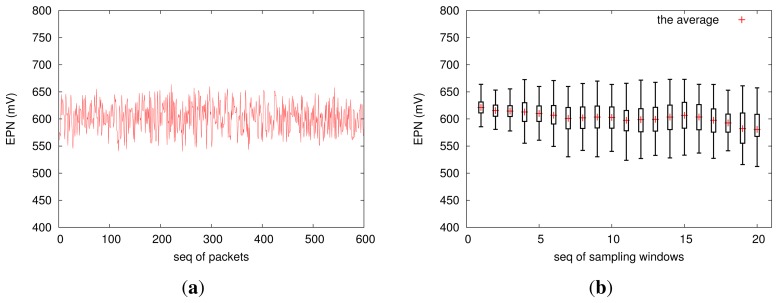
The electrochemical potential noise (EPN) distribution over sampling windows where (**a**) plots the EPN variation during the 10th sampling window; and (**b**) profiles the statistics for each of the 20 consecutive sampling windows.

Figure 10 shows the distribution of EPN, which reflects the dynamics of the electrochemical potential noise (EPN). In fact, EPN data is one of the most important data sources for analyzing pitting corrosion behavior; we will not discuss how to make decisions based on the EPN data, which is beyond the scope of this study. In the future, we will integrate a high-accuracy current-monitoring circuit into EPS, such that it will also capture the electric current output by our corrosion-sensing device.

## 6. Conclusion and Future Work

In this paper, we have designed and implemented EPS, a wireless corrosion monitoring platform using wireless sensor motes powered by micro-corrosion energy. To the best of our knowledge, this is the first comprehensive attempt at harvesting electrochemical environmental energy to power a COTS wireless sensor mote. EPS is an integrated hardware-software system with new designs for a corrosion-sensing device, a supercapacitor-based power supply component and an adaptive transmitted power control. EPS can automatically manage the recharging and discharging of its power supply, with two bounds that are able to be configured by the application; and particularly, EPS uses an adaptive transmitted power control scheme to save energy in communications, thereby prolonging the system lifetime. Besides the comprehensive design considerations, we have also conducted a set of preliminary test-bed experiments; the results demonstrate the feasibility and the energy efficiency of EPS. This study considers only the steel corrosion in RC structures, because steel is most commonly used in diverse structures, such as buildings, bridges, cargo ships, sea drilling platforms, *etc.* We believe that the presented designs and implementation considerations are beneficial for monitoring other metal's corrosion, as well. In the future, we will first integrate an energy-efficient circuit for monitoring corrosion current, providing richer data for field experts, and then, we will extend EPS's control policies to make it suitable for operating in the corrosion-monitoring sensor network deployed for large-scale practical RC structures.
